# GLP-1 Receptor Agonist Exposures Are Increasingly Common and Generally Associated with Mild Symptoms: A Single Poison Center Experience

**DOI:** 10.1007/s13181-024-01008-x

**Published:** 2024-06-11

**Authors:** Stacy Marshall, Erin Ryan, Jessica Rivera, Lindy Reynolds, Suhkshant Atti

**Affiliations:** 1 Department of Emergency Medicine, University of Alabama at Birmingham, Birmingham, Alabama, United States; 2 Alabama Poison Information Center, Children’s of Alabama, Birmingham, Alabama, United States; 3 Department of Pharmacy Practice, University of Illinois Chicago, Chicago, Illinois, United States; 4 School of Public Health, University of Alabama at Birmingham, 203 General Service Building 521 19th Street South, Birmingham, AL 35233 United States

**Keywords:** Glucagon-like peptide-1 Receptor Agonist, Hypoglycemia, Overdose

## Abstract

**Introduction:**

Glucagon-like peptide-1 receptor agonist use has increased over the last decade for glycemic control in type 2 diabetes mellitus, cardiovascular risk reduction, and weight loss. Clinical trials indicate that gastrointestinal adverse effects are commonly experienced and severe hypoglycemia is rare; however, there is little data regarding glucagon-like peptide-1 receptor agonist in overdose.

**Methods:**

We performed a retrospective chart review evaluating and characterizing glucagon-like peptide-1 receptor agonist exposures reported to a single poison center between 2006 and 2023. Patient demographics, circumstances of exposure, clinical effects, and outcomes were abstracted from charts. Descriptive statistics were utilized to summarize demographic information and clinical factor data.

**Results:**

A total of 152 charts met inclusion criteria. Therapeutic errors accounted for 91% of exposures. Most patients (67%) reported no symptoms, although not all patients were followed to a definitive outcome. Nausea, vomiting, generalized weakness, and abdominal pain were the predominant symptoms reported. Most patients (62%) were monitored and closely followed in the home setting. Hypoglycemia was rare but occurred in the setting of a single agent glucagon-like peptide-1 receptor agonist exposure in two patients. Two additional patients who developed hypoglycemia involved co-administration of insulin. 21% of the exposures were related to errors on initial use of the pen.

**Conclusion:**

Exposures to glucagon-like peptide-1 receptor agonist have increased substantially over the years. Effects from an exposure tended to be mild and primarily involve gastrointestinal symptoms. Hypoglycemia was rare. Therapeutic and administration errors were common. Education on pen administration may help to reduce errors.

## Introduction

Glucagon-like peptide-1 receptor agonists (GLP-1 RAs) are a class of medication that mimic the effects of glucagon-like peptide-1 (GLP-1), an endogenous, gut-derived incretin that is released in response to a meal [1]. GLP-1 receptors are located in the pancreas, kidney, stomach, heart, adipose tissue, and brain [2]. Pancreatic β-cell GLP-1 receptor agonism leads to glucose-dependent release of insulin, but this does not solely account for its ability to decrease postprandial glucose concentrations. GLP-1 receptor agonism additionally slows gastric emptying, decreases glucagon secretion, and promotes satiety [3].

Table 1 describes the currently available GLP-1 RAs. Patients are commonly prescribed subcutaneous injections, but a daily, oral formulation of semaglutide now exists. Initially approved for glycemic control in patients with diabetes, selected GLP-1 RAs are also known to aid in weight loss by increasing satiety. These medications are increasingly being prescribed to treat obesity, off-label, though liraglutide, semaglutide, and tirzepatide were approved by the United States Food and Drug Administration (FDA) for this purpose in 2014, 2021, and 2023, respectively. Concordantly, GLP-1 RA exposures reported to poison control centers have increased dramatically in recent years from 241 single substance exposures in 2016, the first year the class was recorded separately in the National Poison Data System, to 2170 exposures in 2022 [4, 5].

**Table 1:** Available glucagon-like peptite-1 receptor agonists marketed in the United states (March, 2023)

Agent	Brand Name	Dose Range	Route of Administration	Frequency	How supplied	Tmax	Elimination Half-life	Protein binding
dulaglutide	Trulicity™	0.75 mg-4.5 mg	subcutaneous injection	weekly	Single-dose pens	48 h	5 days	N/A
exenatide	Byetta®	5 mcg − 10 mcg	subcutaneous injection	twice daily	Multi-dose prefilled syringes	2.1 h	2.4 h	N/A
	Bydureon™	2 mg	subcutaneous injection	weekly	Single-dose pens	2 weeks; 2nd peak at 6–7 weeks		N/A
liraglutide	Saxenda® Victoza®	0.6 mg-3 mg	subcutaneous injection	daily	Multi-dose prefilled syringes	8–12 h	12.3–13 h	55%
semaglutide	Ozempic®	0.25 mg-2 mg	subcutaneous injection	weekly	Multi-dose prefilled syringes	1–3 days	1 week	> 99% (plasma albumin)
	Wegovy™	0.25 mg-2.4 mg	subcutaneous injection	weekly	Single-dose pens	1–3 days	1 week	> 99% (plasma albumin)
	Rybelsus®	3 mg-14 mg	oral	daily	Tablets	1 h	1 week	> 99% (plasma albumin)
tirzepatide	Mounjaro™	2.5 mg-15 mg	subcutaneous injection	weekly	Multi-dose prefilled syringes	8–72 h	5 days	99% (plasma albumin)

*Abbreviations*: mg: Milligram; Tmax: Time to peak drug concentration

At therapeutic doses, gastrointestinal side effects are common for all GLP-1 RAs. Gastrointestinal symptoms developed in 56% of participants in one trial [6]. The risk of hypoglycemia (blood sugar </= 70 mg/dL) is theoretically lower than other antidiabetic agents given insulin is released in a glucose-dependent manner. This low risk is further supported by clinical trial data reporting a low incidence (1–2%) of severe or major hypoglycemia, often defined as blood glucose < 56 mg/dL, hypoglycemia requiring therapeutic intervention, and/or hypoglycemia with severe symptoms [7]. Minor hypoglycemia, which is also not universally defined by available clinical trials but typically involves symptoms of hypoglycemia in which the patient self-treated, is reported at a higher incidence of 0-15.9% [7]. The rate of minor hypoglycemia has been shown to increase as high as 42% with concomitant sulfonylurea and/or insulin therapy [7, 8].

Data regarding adverse effects of GLP-1 RAs in overdose are sparse and largely limited to case reports of intentional and supratherapeutic exposures. Published cases primarily describe gastrointestinal symptoms and episodes of relative hypoglycemia with patients receiving supportive therapies when admitted to the hospital. Currently, there is no consensus on observation time or management of patients who present after an acute overdose of a GLP-1 RA. We present a summary of GLP-1 RA exposure and overdose data obtained from a single poison center over an 18-year period.

## Methods

This is a retrospective analysis of GLP-1 RA exposures reported to a single poison center between January 1, 2006 and March 2, 2023. Poison center records were queried for all patients in which a GLP-1 RA was listed as an exposure. Charts were divided equally among all authors, which included medical and clinical toxicologists and a certified specialist in poison information experienced in the electronic databases used, thus no additional formal training was required. Clinical definitions were agreed upon by all reviewers prior to data abstraction and consensus was reached regarding interpretation of variables and case coding. Duplicate charts and charts in which no GLP-1 RA exposure occurred were excluded. Patient demographics, route of administration, circumstances of exposure, management location, observation time, clinical effects, medical and non-medical interventions, and outcomes were abstracted from the electronic medical record. Further, case narratives documented by specialists in poison information were reviewed to ensure accuracy of extracted data. Discussion among authors occurred in any case where a discrepancy between a case narrative and coded data was discovered with case narratives taking precedence over inaccurate coding. Hypoglycemia was defined as a blood glucose < 70 mg/dl at any point after the exposure. Acute kidney injury was defined as serum creatinine (SCr) > 1.2 mg/dl or an increase of > 0.3 mg/dl from baseline and acute liver injury as AST > 48 U/L or ALT > 55 U/L not attributable to another etiology. Demographic and clinical factors were summarized using descriptive statistics, and relevant hypothesis testing was conducted as indicated. Fisher exact test was performed to determine if there were any differences in symptom frequency among GLP-1 RAs. All analyses were conducted in SAS 9.4 (Cary, NC, USA). Microsoft Excel was used for figures. This study was reviewed by the local institutional review board and determined to be exempt.

## Results

**Fig. 1:**
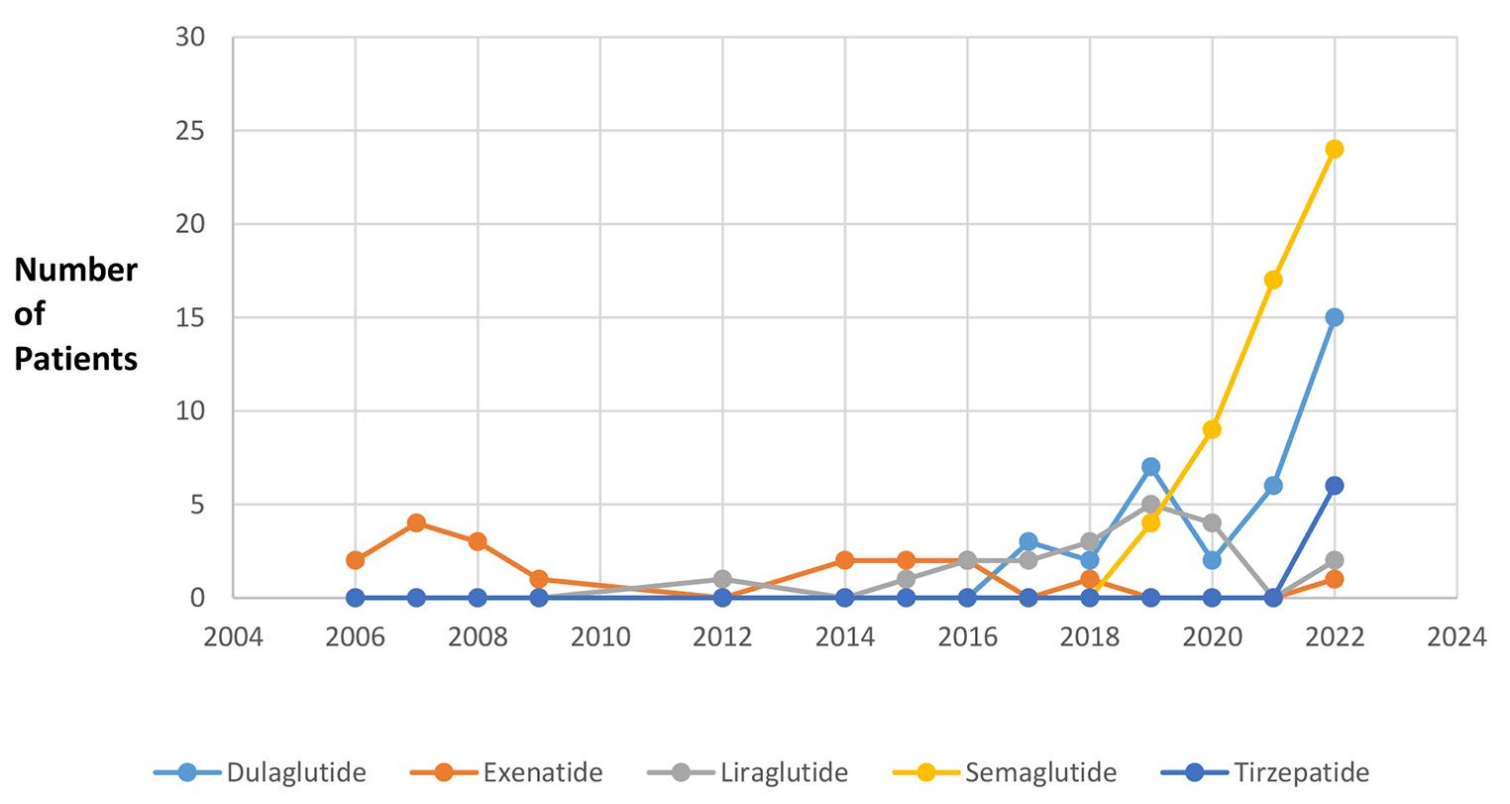
Exposures by individual agent each year between 2006–2022 *data from 2023 were excluded as the data was only collected up until March 2023

A total of 166 charts were identified; 152 charts met inclusion criteria. Demographics information are reported in Table 2. Most exposures occurred in females (*n* = 116, 76%). The median age was 53 years (range 2 to 94 years). Fewer than seven exposures were reported each year between 2006 and 2018, with a sharp increase in 2019 (*n* = 16) and a peak in 2022 (*n* = 48) (see Fig. 1). A history of diabetes mellitus was reported in 107 (70%) patients. 10% of patients (*n* = 16) clearly indicated the medication was being used for weight loss, but indication of prescription was not reported in 38% (*n* = 58) of patients. Specific agents involved in exposures included semaglutide (*n* = 65, 43%), dulaglutide (*n* = 38, 25%), liraglutide (*n* = 22, 14%), exenatide (*n* = 18, 12%), and tirzepatide (*n* = 9, 6%). In this cohort, subcutaneous injection (*n* = 141, 93%) was the primary route of exposure, followed by ingestion of oral tablets (*n* = 10, 7%). Of those 10 patients with oral exposures, most (*n* = 5, 50%) resulted from inadvertently taking a double dose and only 1 (10%) patient manifested symptoms (fatigue and nausea). Patients were otherwise asymptomatic and 90% (*n* = 9) were monitored at home with an average length of 8 h (range 1–21 h) of poison center follow up. No patient with an oral exposure required referral to a healthcare facility. Most patients reported single substance exposures (*n* = 146, 96%) but concomitant antihyperglycemic medication use was recorded in 51 patients (34%). Fifty patients (33%) reported taking at least one antidiabetic agent that was not an antihyperglycemic agent (e.g. metformin).

**Table 2:** Exposures to GLP-1 receptor agonists reported to the Alabama Poison Information Center, January 1, 2006 - March 2, 2023

	All GLP-1 RAs (*N* = 152)	Dulaglutide (*n* = 38)	Exenatide (*n* = 18)	Liraglutide (*n* = 22)	Semaglutide (*n* = 65)	Tirzepatide (*n* = 9)
Age (Years) (Mean ± SD)	51.5 ± 16.1	52.9 ± 19.5	54.5 ± 6.5	52.7 ± 16.4	50.6 ± 15.8	42.7 ± 15.6
Sex n (%)						
Female Male	116 (76) 36 (24)	21 (55.3) 17 (44.7)	16 (88.9) 2 (11.1)	16 (72.7) 6 (27.3)	54 (83.1) 11 (16.9)	9 (100) 0 (0.0)
Weight (Kg) (Mean ± SD)	98.1 ± 31.3	93.6 ± 35.7	113.8 ± 47.1	109.5 ± 33.9	92.6 ± 28.7	102.9 ± 22.9
Hx of CKD n (%)	3 (0.02)	0 (0.0)	0 (0.0)	0 (0.0)	3 (0.05)	0 (0.0)
Hx of DM n (%)	107 (70.4)	27 (71.1)	17 (94.4)	19 (86.4)	43 (66.2)	1 (11.1)
Other Past Medical History	129 (84.9)	32 (84.2)	17 (94.4)	20 (90.9)	53 (81.5)	7 (77.8)
Prescribed Dose (mg) Mean (SD)		1.47 (± 0.94)	0.26 (± 0.68)	1.35 (± 0.51)	1.86 (± 3.57)	5.62 (± 3.75)
Indication for Rx n (%)						
Diabetes Weight Loss Not Patient’s Rx Unknown	65 (42.8) 16 (10.5) 13 (8.6) 58 (38.2)	13 (34.2) 1 (2.6) 7 (18.4) 17 (44.7)	16 (88.9) 0 (0.0) 0 (0.0) 2 (11.1)	14 (63.6) 3 (13.6) 0 (0.0) 5 (22.7)	21 (32.3) 7 (10.7) 4 (6.2) 33 (50.8)	1 (11.1) 5 (55.6) 2 (22.2) 1 (11.1)
Taking antidiabetic, non-hypoglycemic medications n (%)	50 (32.9)	13 (34.2)	7 (38.9)	7 (31.9)	21 (32.3)	2 (22.2)
Taking Other Hypoglycemic medications n (%)	51 (33.6)	12 (31.6)	8 (44.4)	15 (68.2)	16 (24.6)	0 (0.0)

*Abbreviations*: CKD: Chronic kidney disease; DM: Diabetes mellitus; GLP-1 RA: Glucagon-like peptide-1 receptor agonist; mg: milligrams; Rx: Prescription; SD: Standard deviation

Almost all exposures were unintentional (*n* = 143, 94%). Three patients (2%) had injected the product in a suspected self-harm attempt. Of those 3, one patient had a previous diagnosis of bipolar disorder. No previous psychiatric diagnoses were documented in the remaining two. Forty patients (26%) administered a double dose within an hour of administration of the first dose. Many of these involved first time use error secondary to misunderstanding of how to use the injection pen (*n* = 32, 21%). In this study, pen associated errors were most reported with semaglutide (35%); of note, semaglutide was also the most frequent medication exposure in this study (*n* = 65, 43%). Additionally, 25 patients (16%) reported double dosing within 24 h of the first dose and 12 (8%) patients reported injecting a once weekly formulation daily. Notably in the latter cohort, 2 patients injected weekly formulations daily for 12 and 14 days and one patient injected semaglutide 0.5 mg twice a day for 1 month. No patient who injected a weekly formulation daily required hospital admission.

While most patients reported no symptoms (*n* = 107, 67%), it is worth noting that many (*n* = 65, 43%) were lost to follow-up and that most subcutaneous injection exposures remained within a normal therapeutic dosing range (Table 3). Adverse effects included nausea (*n* = 28, 18%), vomiting (*n* = 18, 11.8%), ‘weakness’ (n = 16, 10%), and abdominal cramping (n = 8, 5%) as referenced in Table 4. No differences were observed between the agent involved and the symptoms experienced, however tirzepatide and liraglutide exposures resulted in higher incidence of nausea (33.3% and 31.8%, respectively). Patients experiencing nausea with other agents ranged from 11.1%-15.8%. Acute liver injury, acute kidney injury, and pancreatitis were not reported in any patients. Four patients (2.6%) developed hypoglycemia (blood glucose range 40–62 mg/dL). Two of these were self-harm attempts in which one patient injected 4 semaglutide pens of unknown concentration and 100 units of long-acting insulin. The second patient injected a full dulaglutide pen of unknown concentration along with 100 units of long-acting insulin. Both patients were admitted to the intensive care unit (ICU) and received continuous dextrose infusions for 3 days. A third patient mistakenly injected 40 mg of semaglutide instead of insulin and had resolution of hypoglycemia within 24 hours after a dextrose bolus and feeding. A fourth patient intentionally injected the maximum dose of 4.5 mg of dulaglutide, which was not prescribed to her, to “induce ketosis”. This was the only patient within this cohort to exhibit hypoglycemia after a therapeutic dose, albeit the maximum dose. Seven patients (4%), including 3 of the 4 patients with hypoglycemia, were initiated on continuous dextrose infusions. Indications, infusion rates, and duration of therapy were not consistently reported or documented.

**Table 3:** Reported dose of GLP-1 RA administered and therapeutic dose range

GLP-1 Agonist	Median Dose of Exposure (mg)	Interquartile Range	Therapeutic Dose Range
Dulaglutide	3.00	1.50–4.50	Trulicity™: 0.75–4.5 mg
Exenatide	0.02	0.015–0.020	Byetta®: 0.005–0.010 mg
Liraglutide	3.60	2.40–3.60	Saxenda®, Victoza®: 0.6 -3 mg
Semaglutide	2.00	1.00–4.00	Ozempic®: 0.25–2 mg Wegovy™: 0.25–2.4 mg Rybelsus®: 3–14 mg
Tirzepatide	7.50	2.50–10.0	Mounjaro™: 2.5–15 mg
Exenatide (ER*)	4.00	4.00–4.00	Bydureon™: 2 mg

*ER = Extended Release

**Table 4:** Common symptoms reported in GLP-1 RA exposures

Clinical Effect	All GLP-1 RA (*N* = 152)	Dulaglutide (*n* = 38)	Exenatide (*n* = 18)	Liraglutide (*n* = 22)	Semaglutide (*n* = 65)	Tirzepatide (*n* = 9)	*p*-value^1^
Generalized Weakness n (%)	16 (10.5)	5 (13.2)	2 (11.1)	3 (13.6)	5 (7.9)	1 (11.1)	0.72
Nausea & Vomiting n (%)	28 (18.4)	6 (15.8)	2 (11.1)	7 (31.8)	10 (15.4)	3 (33.3)	0.40
Abdominal pain or cramping n (%)	8 (5.3)	3 (7.9)	0 (0.0)	1 (4.5)	4 (6.2)	0 (0.0)	0.54
Diarrhea n (%)	2 (1.3)	0 (0.0)	0 (0.0)	0 (0.0)	2 (3.1)	0 (0.0)	0.50
Loss of Appetite n (%)	2 (1.3)	1 (2.6)	0 (0.0)	1 (4.5)	0 (0.0)	0 (0.0)	0.28
Other Adverse Effects n (%)	8 (5.3)	2 (5.3)	3 (7.9)	1 (4.5)	2 (3.1)	0 (0.0)	0.27
Hypoglycemia n (%)	4 (2.6)	2 (5.3)	0 (0.0)	0 (0.0)	2 (3.1)	0 (0.0)	0.55

*Abbreviations*: GLP-1 RA: Glucagon-like peptide-1 receptor agonist

^**1**^Fisher’s exact test

Patients in this cohort were primarily monitored at home (*n* = 94, 62%); however, 5 patients (3%) required ICU level of care for frequent glucose monitoring, 7 (5%) were admitted to the floor, and 22 (14%) were discharged from the ED. The remaining 25 patients (16%) were lost to follow-up (Table 5). Patients requiring hospital admission had exposures involving subcutaneous administration and the majority (83%) involved a single-substance exposure. However, 6 of the 12 admitted patients were exposed to supratherapeutic doses, while 4 patients reportedly injected therapeutic doses. Additionally, 3 of the 12 admitted patients were concomitantly taking insulin and 1 patient was on sulfonylurea therapy. Median hospital stay was 2 days (range, 1–4 days) and median ED stay was 5 h (range, 2–120 h). Patients managed on-site were followed for a median of 4.5 h (range, 0–120 h).

**Table 5:** Management site and length of observation

Management Site	Number (%)	Length of Observation (Hours) Median (range)
Home	94 (62%)	4.5 (0-120)
Intensive Care Unit	5 (3%)	48 (24–96)
Floor	7 (5%)	48 (24–96)
Discharged from emergency department	22 (14%)	5 (2-120)

Pediatric exposures were uncommon in this cohort. Five pediatric patients were reported to the poison center during the study period with an age range of 2–12 years. Both oral (*n* = 2) and subcutaneous exposures (*n* = 3) were reported. No pediatric patient developed symptoms or hypoglycemia. Exposure dose was unknown in most patients. Two pediatric patients were admitted to the hospital for close monitoring, but neither required intervention.

## Discussion

While the overall number of GLP-1 RA exposures increased during the study period, outcomes were largely benign, with the most common being no effect. Only seven patients (5%) experienced moderate effects, and no major effects or deaths were recorded. Most patients were managed on-site with poison center follow-up and not referred into a healthcare facility, likely due to the large number of double doses that remained within a therapeutic dosing range (Table 3). In patients who became symptomatic, gastrointestinal effects predominated. These effects are known to occur frequently even with therapeutic use of GLP-1 RAs; between 10 and 56% of patients in clinical trials complained of nausea, vomiting, and/or diarrhea [8]. Post-marketing data and pharmacovigilance registries have suggested a possible association between GLP-1 RA and acute pancreatitis although this remains controversial [9–12]. Post-marketing reports of acute pancreatitis, including both cases of necrotizing and hemorrhagic pancreatitis, associated with exenatide prompted the FDA to require a product label warning which now extends to all newly marketed GLP-1 RAs. Pancreatitis was not reported in this relatively small cohort. Adverse effects with supratherapeutic doses and therapeutic errors appear to be largely an extension of those seen with therapeutic use, and the majority of unintentional exposures to GLP-1 RAs can likely be safely monitored at home.

Hypoglycemia is a concern in many exposures to antidiabetic agents, however low blood glucose was a rare finding in this study. While four patients developed hypoglycemia, two of these patients co-administered large doses of a long-acting insulin. Insulin’s additive effect makes it challenging to determine the extent the GLP-1 RA contributed to the hypoglycemia. Three of the four patients who developed hypoglycemia had clearly taken supratherapeutic doses of the GLP-1 RA, while one patient had taken the maximum recommended dose of the GLP-1 RA, but this medication was not prescribed to her. In this cohort, hypoglycemia was rare in patients who had a therapeutic exposure. No pediatric patient developed symptoms or hypoglycemia. However, the small number (*n* = 5) of pediatric patients and missing dosing information in most instances limit generalizability to the pediatric population. No patient in this cohort who developed hypoglycemia reported concomitant use of a sulfonylurea. Previous studies have suggested that therapeutic GLP-1 RAs given in combination with agents that lower blood glucose may increase the risk of hypoglycemia [13]. Exposures involving these agents in addition to sulfonylureas or insulin may warrant closer monitoring.

Overall, it appears that significant hypoglycemia is unlikely in single-substance exposures, even in large overdoses. This is consistent with the known mechanism of these medications which enhance insulin secretion only in a glucose-dependent manner [8]. Previously published case reports of intentional and unintentional supratherapeutic exposures have mostly described gastrointestinal symptoms requiring hospital admission for supportive therapies, such as intravenous fluids and antiemetic medications [14–21]. Of these case reports, no episodes of overt hypoglycemia occurred, however episodes of relative hypoglycemia were reported. One published case involving a non-diabetic patient with an intentional, single-exposure overdose on liraglutide depicts severe hypoglycemia requiring dextrose infusion and octreotide [22].

Therapeutic errors accounted for 91% of exposures during the study period. The most common scenarios were double doses and doses taken too closely together. A common reason given for the error was confusion surrounding how to use pen devices by which these agents are administered. Patients in the current study frequently reported believing the pen had malfunctioned on initial dosing when it had not, and then gave an additional dose before realizing their error. Many of the subcutaneous products are administered once weekly, which contributes to medication errors, as demonstrated in this study, where 8% of patients reported injecting the weekly medication daily. Patients are likely to be unfamiliar with, and are often offered little training, on the use of subcutaneous administration of medications, which can contribute to medication errors. A previous volunteer study assessing accurate use of three different GLP-1 RA injection devices found that between 17 and 57% of patients made errors when injecting a dose for the first time [23]. This suggests there is a need for thorough counseling on pen use at the time of dispensing. This risk may be further amplified with the increase in compounded semaglutide products. The FDA has issued warnings about adverse effects related to improperly compounded GLP-1 RAs as shortages force patients to seek alternative supplies. Unfortunately, the source of the product was not recorded in this cohort, so it is unclear whether compounding issues played a role in any adverse events seen.

Intentional overdose exposures are worth noting in this study although limited conclusions can be made given the small sample size (*n* = 3). Intentional overdoses in this study involved dulaglutide (*n* = 2) and semaglutide (*n* = 1). The European Medicines Agency (EMA) has recently reported trends of patients having suicidal thoughts and self-injury associated with GLP-1 RA use, most specifically liraglutide and semaglutide [24]. Similarly, reports of suicidal behavior associated with GLP-1 RA exposure have been reported to the FDA Adverse Event Reporting System (FAERS) [25, 26]. In a study by McIntyre et al. (2023) evaluating FDA FAERS data, suicidal ideation, suicide attempts, and completed suicide were also disproportionately associated with liraglutide and semaglutide; the same was not found for other GLP-1 RAs. The potential link between use of GLP-1 RA and suicidality is unknown but likely not due to one specific drug mechanistic effect and complicated by many confounding factors. For example, there is a known association between type 2 diabetes, obesity, dieting, rapid weight fluctuations, and mental health disorders such as depression, anxiety, and body dysmorphia [26]. In addition to areas such as the pancreas, GLP-1 receptors are also located in the brain such as the hypothalamus, nucleus accumbens, hippocampus, and amygdala and GLP-1 RAs also interact with serotonin, dopamine, and glutamate neurotransmitter systems. All aforementioned systems are important in regulating emotions, impulsive behaviors and reward pathways [25]. Finally, given that GLP-1 agonists have become mainstream and popular in the media, it may just be that reporting of adverse events is higher relative to other drugs [26].

GLP-1 RA exposures were rare at this center until 2019, consistent with national trends. Data collected in the first three months of 2023 suggest that the frequency of exposures may continue to climb. These results parallel an overall expansion of prescribing of these medications over the past decade as evidence has accumulated supporting improved cardiovascular and renal outcomes in addition to their benefits in glycemic control and weight loss [27–29]. Poison centers should expect continuing increases in reports as these agents’ indications continue to be expanded and more patients begin taking them. Appropriate education measures including ensuring appropriate counseling on pen use as well as provider education on important drug-drug interactions may be of public health concern as this trend continues.

## Limitations

As a retrospective review of data from a single poison center, this study has several limitations. Case records are primarily from self-reported calls to the poison center. Identification of patients relies on accurate substance recognition by the caller and accurate coding at the poison center, and it is possible that other patients exposed to GLP-1 RA agents were not included. Additionally, exposure details are limited to those that were reported to the specialist in poison information at the time of the call and the poison center was unable to verify the accuracy of the report; it is likely that not all symptoms and therapies were recorded in the poison center chart and many patient charts were missing data points. Laboratory values were only available in a minority of patients since most cases were followed in the home setting, so it is possible that the incidence of hypoglycemia was underestimated. Time points, such as time of discharge if at a healthcare facility, were estimated based on times of telephone follow up; this may have led to overestimation of lengths of stay and durations of observation. Additionally, over one third of the exposures were not followed to a definitive outcome whether due to follow-up being deemed unnecessary or the patient being lost to follow up. Because of this, there is also a possibility that patients coded as no effect or minor effect may have developed symptoms after the final follow-up and symptoms were under-recorded. Finally, these data are from a single regional poison center, and for that reason the generalizability of our findings to other regions are limited.

## Conclusions

Exposures to GLP-1 RAs reported to a regional poison center increased substantially over the past 18 years, likely due to increased prescribing of these medications as indications for their use have expanded. Consequences of therapeutic errors appear to be mild and primarily involve gastrointestinal symptoms with few patients developing hypoglycemia, especially in the absence of another hypoglycemic agent. Therapeutic errors and administration issues are common, and improved education when these medications are prescribed and dispensed may be of use in reducing overdoses. Opportunity exists to improve how many of these agents are supplied. Providing multi-dose pens and vials may increase the risk for a medication error to occur.
